# Identification of Differential Gene Groups From Single-Cell Transcriptomes Using Network Entropy

**DOI:** 10.3389/fcell.2020.588041

**Published:** 2020-10-22

**Authors:** Yanglan Gan, Shanshan Liang, Qingting Wei, Guobing Zou

**Affiliations:** ^1^School of Computer Science and Technology, Donghua University, Shanghai, China; ^2^School of Software, Nanchang University, Nanchang, China; ^3^School of Computer Engineering and Science, Shanghai University, Shanghai, China

**Keywords:** network entropy, differential genes, protein interaction network, single-cell transcriptome, gene expression

## Abstract

A complex tissue contains a variety of cells with distinct molecular signatures. Single-cell RNA sequencing has characterized the transcriptomes of different cell types and enables researchers to discover the underlying mechanisms of cellular heterogeneity. A critical task in single-cell transcriptome studies is to uncover transcriptional differences among specific cell types. However, the intercellular transcriptional variation is usually confounded with high level of technical noise, which masks the important biological signals. Here, we propose a new computational method DiffGE for differential analysis, adopting network entropy to measure the expression dynamics of gene groups among different cell types and to identify the highly differential gene groups. To evaluate the effectiveness of our proposed method, DiffGE is applied to three independent single-cell RNA-seq datasets and to identify the highly dynamic gene groups that exhibit distinctive expression patterns in different cell types. We compare the results of our method with those of three widely applied algorithms. Further, the gene function analysis indicates that these detected differential gene groups are significantly related to cellular regulation processes. The results demonstrate the power of our method in evaluating the transcriptional dynamics and identifying highly differential gene groups among different cell types.

## 1. Introduction

A complex tissue contains a variety of heterogeneous cell types, each with its own distinct features and function (Guo et al., 2017). Single-cell RNA sequencing (scRNA-seq) has allowed researchers to quantify gene expression at a cellular resolution (Jaitin et al., 2014; Wu et al., 2014). Given the scRNA-seq data of a population of cells, one of the fundamental data analysis tasks involves characterizing cellular heterogeneity and quantifying such substantial variability via differential analysis (Stegle et al., 2015). Gene expression is inherently stochastic, and some intercellular variation arises from transcriptional bursting of individual genes or coordinated fluctuations of multi-gene networks (Soltani et al., 2016). Generally, the objective of differential analysis is to searching for those genes exhibiting significant differences in abundance associated with different cell types (Jaakkola et al., 2017). This is a key step for downstream analysis, such as identifying developmentally regulated genes, understanding the functionality and cell fate (Trapnell et al., 2014).

There are a plethora of approaches that have been proposed to detect differential genes (Wang et al., 2019). Some methods are originally developed for differential expression analysis of bulk RNA-seq data. The widely applied method DESeq adopts a negative binomial distribution model count data, with mean and variance linked by local regression (Anders and Huber, 2010). An overdispersed Poisson model is used in the method edgeR to account for both biological and technical variability (Robinson et al., 2010). Limma is based on linear modeling and has shown good performance in previous comparison studies (Ritchie et al., 2015). As these methods target the detection of differentially expressed genes of overall expression between the populations, they are hard to take full advantage of the rich information provided by single-cell RNA-seq data. Recently, researchers have designed several new methods to detect differential genes from single-cell transcriptomes. SCDE detects differentially expressed genes based a two-part joint model, which can respectively accommodate multi-model expression values and drop-out events (Kharchenko et al., 2014). MAST represents the zero counts and positive expression values by a hurdle model, and further adopts logistic regression and linear regression to identify differential genes for each part (Finak et al., 2015). Monocle2 applies generalized additive models to identify differential genes (Qiu et al., 2017). DEsingle also exploits zero-inflated negative binomial model to estimate the proportion of real and dropout zeros, and to detect three types of differential genes in scRNA-seq data (Miao et al., 2018). As most of the available methods perform a pairwise comparison of single gene, they might neglect the dependencies and information among genes. As previous research show that genes in the same pathway tend to correlate in expression, the network structure of genes can boost differential analysis (Dona et al., 2017). Therefore, to reduce the impact of the noises existed in the scRNA-seq data and increase predictive power, it would be necessary to exploit both the expression levels and the interaction information of these genes.

To address this issue, we propose DiffGE, a new computational method for differential analysis from single-cell transcriptome. As genes in the same pathway tend to exhibit strong co-relationship and have consistent changes in the direction of expression levels, DiffGE adopts network entropy to evaluate the variation of gene groups, which takes the dependencies and interactions of genes into account, and further identifies highly dynamic gene groups across different cell types. We apply DiffGE to three real scRNA-seq datasets from developmental and disease studies. The experimental results demonstrate that DiffGE is effective in evaluating the transcriptional dynamics and identifying highly differential gene groups. It facilitates general comparisons of scRNA-seq data sets, potentially deepening our understanding of how distinct cell states respond to perturbation, disease, and evolution.

## 2. Materials and Methods

### 2.1. Overview of the Proposed DiffGE

**Figure 1:**
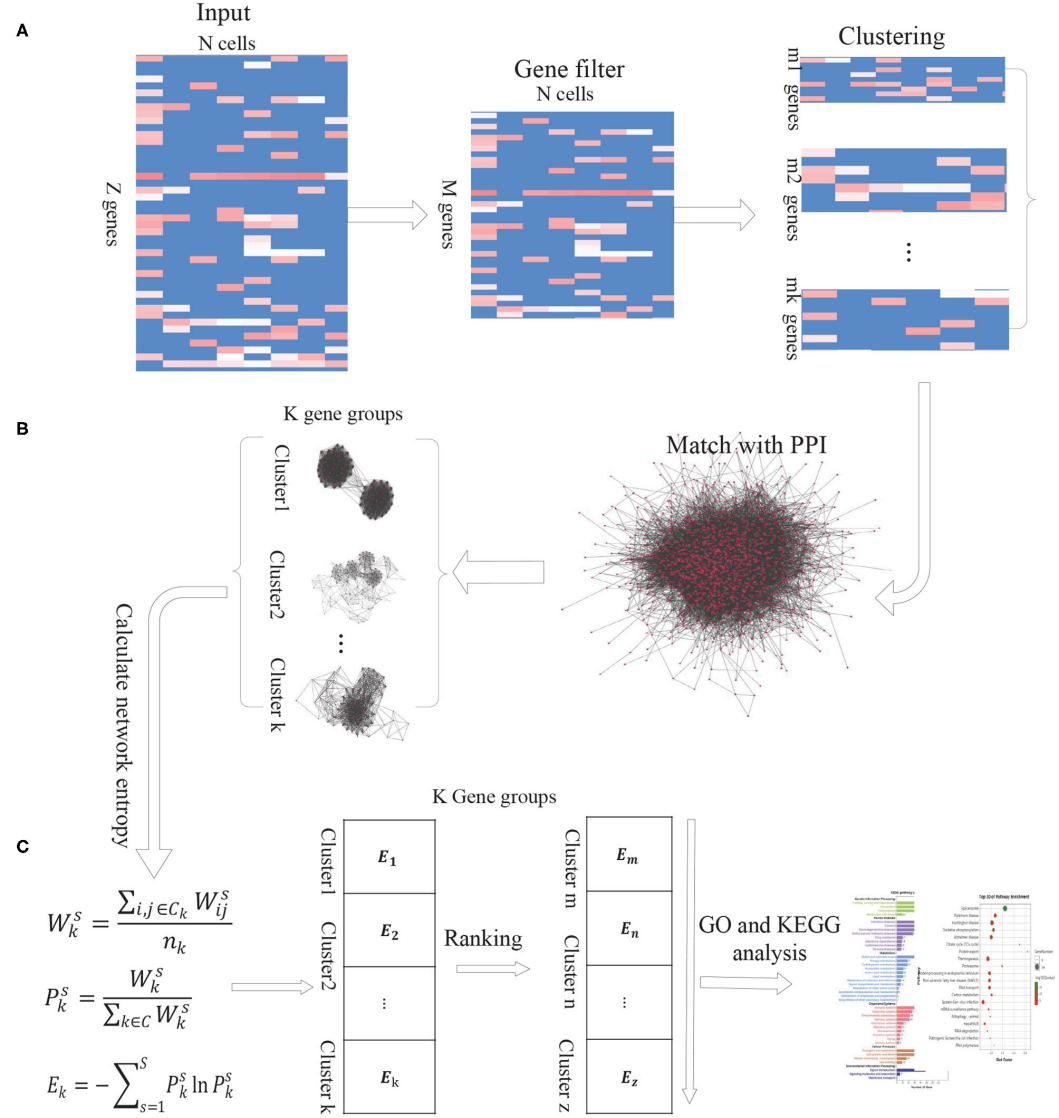
The schematic flowchart of DiffGE. **(A)** Clustering gene based on gene expression matrix. **(B)** Matching gene clusters with PPI network. **(C)** Calculating the entropy of gene clusters on different cells and identifying highly dynamic gene groups.

As the intercellular variation arises from transcriptional bursting of individual genes or coordinated fluctuations of multi-gene networks, we propose a new computational method DiffGE, using network entropy to evaluate the expression variation of gene groups and identifying the significant differential gene groups from scRNA-seq data. As illustrated in Figure 1, the proposed method consists of three main steps. First, clustering genes based on gene expression similarity. Second, matching the identified genes cluster with PPI network. Third, calculating the network entropy of gene clusters across different cell types and identifying the highly differential gene groups. In the following, we describe each step in detail.

#### 2.1.1. Step 1. Clustering Genes Based on Gene Expression Matrix

Let *S* denote a collection of single cells from an scRNA-seq experiment, and *G* denote a set of annotated genes measured in the experiment. *E* represents the gene expression matrix, where *e*_*si*_ ≥ 0 be the expression level of the *i*th gene in the *s*th cell (*i* ∈ *G*, *s* ∈ *S*). Based on the gene expression matrix, we utilize EM clustering method to partition these genes into *K* clusters, which are represented as a collection of *K* subsets of genes, *C* = {*c*_*k*_, *k* = 1, 2, ..., *K*}.

#### 2.1.2. Step 2. Matching the Identified Genes Cluster With PPI Network

For these identified gene clusters, the calculation of their network entropies requires estimating the interaction probabilities among genes. Based on the assumption that two genes will have a greater interaction probability when they are known to interact at the protein level (Banerji et al., 2013), we integrate a comprehensive protein-protein interaction (PPI) network with the gene expression profile of given cell types. We retrieve the protein interaction network from the STRING database, which brings together protein interactions from several distinct sources, including experimental data, computational prediction methods and public literature collections. Then the genes in each gene cluster are matched with the downloaded PPI network. If any two genes have an edge in the corresponding PPI network, the edge is recorded. According to the gene expression profiles of different cells, we can obtain the corresponding PPI-expression networks for each gene cluster.

#### 2.1.3. Step 3. Calculating the Entropy of Gene Clusters on Different Cells

In the PPI-expression network of any gene cluster *k*, if gene *i* is connected to gene *j*, where *e*_*si*_ and *e*_*sj*_ represent the expression levels of gene *i* and gene *j* in the cell *s*, respectively. Similar with previous publications (Guo et al., 2017; Teschendorff and Enver, 2017), the network entropy is calculated as below:

First, the normalized gene expression values of a single cell (or a cell type) are used to assign weights to the edges of the PPI-expression network. Specifically, for a given gene neighbor *j* of gene *i* in the network, we define the edge weight by the product:

$$W_{ij}^{s}=e_{si}*e_{sj}$$

We interpret these weights as interaction probabilities between neighboring genes. In a cell with high expression of gene i and gene j, two genes are more likely to interact than those with low expression of i and/or j (Teschendorff and Enver, 2017). Based on the weights, the activity value of gene cluster *k* on cell *s* is calculated by:

$$W_{k}^{s}=\frac{\sum_{i,j\in C_{k}}W_{ij}^{s}}{n_{k}}$$

Where *C*_*k*_ is the gene cluster *k*, and *n*_*k*_ is the number of genes in gene cluster *k*. The resulted $W_{k}^{s}$ represents the activation probability of the gene group *k* in cell *s*. The more genes of group *k* expressed in cell *s*, the higher the probability that the gene group is activated by the gene expression in *s*.

Then the network entropy for gene cluster *k* among different cells, denoted by *E*_*k*_, is computed according to

$$P_{k}^{s}=\frac{W_{k}^{s}}{\sum_{k\in C}W_{k}^{s}}$$

$$E_{k}=-\overset{S}{\underset{s=1}{\sum }}P_{ks}\ln P_{ks}$$

Where $P_{k}^{s}$ is the ratio of gene cluster *k* to the sum of the activity values of all gene clusters in cell s, and C represents the set of gene clusters.

Based on this model, the network entropy gives an average measure of expression variation in these single cells. Highly differential and plastic gene groups would be characterized by a state of high network entropy. For those gene groups, we sort the calculated network entropies in a descending order and select the gene clusters with significantly higher network entropies as highly differential gene groups. To obtain a proper number of gene clusters, we choose the top 10% of the gene clusters for further analysis.

### 2.2. Evaluation Metrics

To evaluate the performance of our proposed method, we adopt two different metrics. The first measurement is to evaluate the accuracy using receiver operating characteristic (ROC) curves. The area under ROC curve (AUC) is calculated by the R package ROCR. The second metric is to measure the precision and the recall rate of detecting differential genes, which are calculated as the previous study (Jaakkola et al., 2017). Let *DE*_*full*_ be the set of detected differential genes in the full data set, and *DE*_*subset*_ be the set of detected differential genes in a subset of the data. The precision and the recall are respectively defined as:

$$Precision=\frac{DE_{full}\cap DE_{subset}}{DE_{subset}}$$

$$Recall=\frac{DE_{full}\cap DE_{subset}}{DE_{full}}$$

where ∩ denotes in the intersection between two sets. If the number of differential genes detected in the subset is 0, the precision is considered as missing.

### 2.3. Datasets

To evaluate the performance of the proposed DiffGE for differential expression analysis in scRNA-seq data, we apply it to three independent real scRNA-seq datasets from both Homo sapiens and Mus musculus. In detail, the first scRNA-seq data set is from the study by Itay Tirosh et al. The single-cell transcriptome that was sequenced in this study is available at GEO under the accession number GSE72056 (Tirosh et al., 2016). The data set includes gene expression profiles from both malignant and benign cells of melanoma. Specifically, the benign cells consist of six different subtypes of cells. The second scRNA-seq dataset, originated from a study by Islam et al. (2011), contains 48 mouse embryonic stem cells and 44 mouse embryonic fibroblasts. It is available in GEO database under accession number GSE29087. The third scRNA-seq dataset (GSE59114) is from a study by Kowalcyk et al., which consists of long term hematopoietic stem cells (LTHSC) (Kowalczyk et al., 2015).

The expression data transformed by logTPM are used as inputs of different methods. Since the number of genes in the data set is much larger than the number of cells, we utilize two types of gene filters for these scRNA-seq datasets (Jaakkola et al., 2017). First, in order to alleviate the effect of drop-out events on the following analyses, the genes with zero read count in all cells are firstly filtered out (Ji and Ji, 2016). Meanwhile, as the ubiquitous and rare genes are not informative, they are filtered out for further analysis. In detail, if the genes are expressed in less than *v*% of cells, they are regarded as rare gene. Ubiquitous genes are defined as those that are expressed in at least (*100-v*)% of cells. Here, as the previous study (Kiselev et al., 2017), we set *v* as 10.

## 3. Results

### 3.1. Performance Comparison

To validate the utility of DiffGE, we compare it with three widely used methods, including Limma, MAST and Monocle2. Specifically, Limma is based on linear modeling and has shown good performance on bulk RNA-seq data (Ritchie et al., 2015). MAST and Monocle2 are proposed for the analysis of scRNA-seq data. MAST adopts logistic regression and linear regression to identify differential genes for each part (Finak et al., 2015). Monocle2 utilizes reversed graph embedding to sort cells and analyze differential gene expression (Qiu et al., 2017). These methods are applied to two different scRNA-seq data sets, to test their ability of detecting differential genes among different cells. These differential expression methods are tested following the instructions and recommendations of their respective software packages. At the same time, for a fair comparison, we vary different values of parameters for these compared methods and reported the best results.

**Figure 2:**
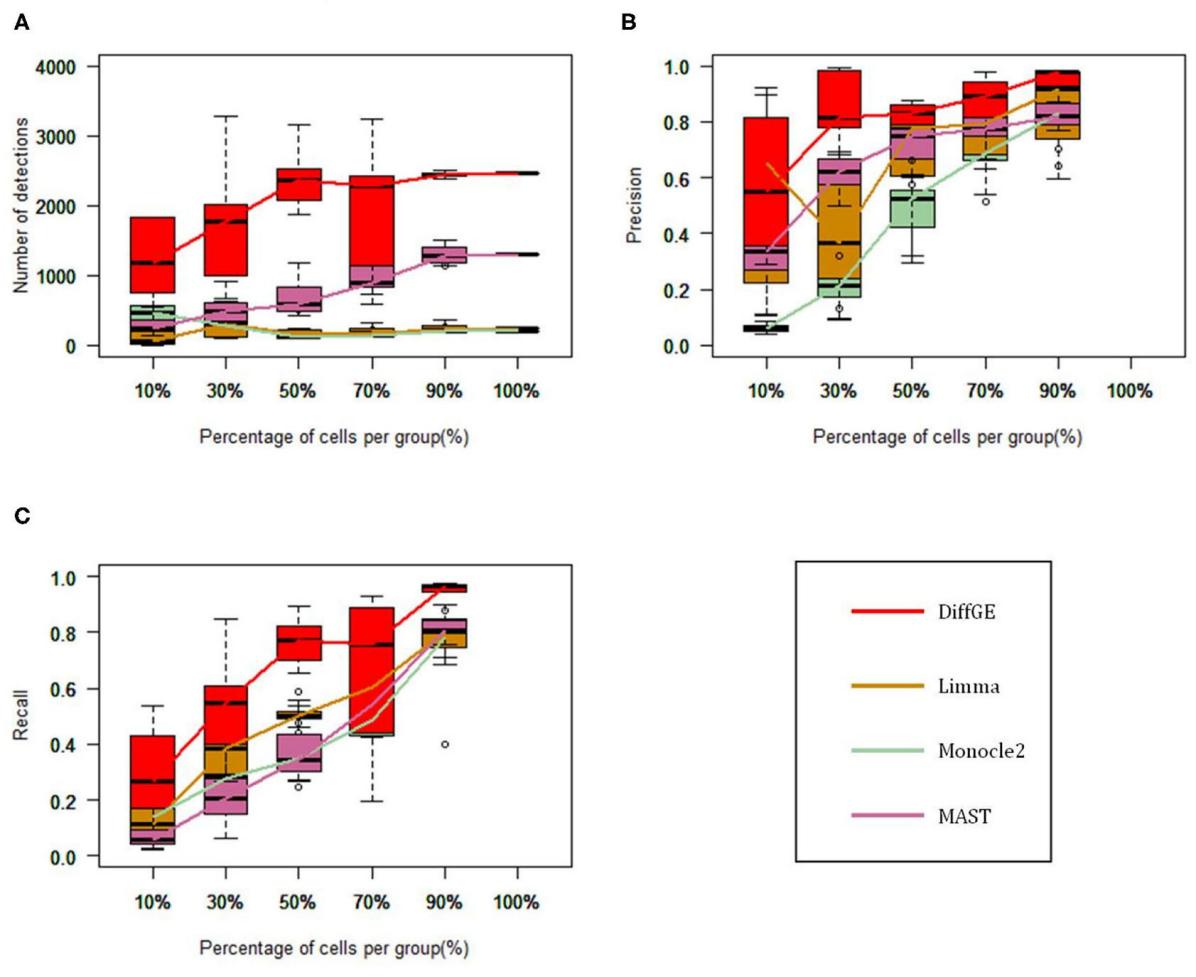
Performance comparison among DiffGE, Limma, MAST, and Monocle2 on the LTHSC datasets. **(A)** Number of detected differential genes. **(B)** Precision. **(C)** Recall rate.

We first test these algorithms on the LTHSC data set, which is a real single-cell dataset from the study by Kowalczyk et al. Cells with very low expression rates are filtered, and we focus on a total of 89 cells from young mice and 120 cells from old mice (Kowalczyk et al., 2015). As in the comparison study (Jaakkola et al., 2017), to estimate the accuracy of these methods, we consider the genetic data detected from the complete data as the gold standard. We respectively extract 10, 30, 50, 70, and 90% of the total cells to form a subset, and each subset is randomly selected and repeated 10 times. We then compute the precision and recall rate. Figure 2 show the performance of the four methods on different subsets. Figure 2A illustrates the numbers of the identified highly differential genes of different methods. Overall, DiffGE detects the largest number of differential genes. Limma and Monocle2 have similar smaller numbers of detections. As the number of cells in the subgroup increases, the number of detections increases. The reason might be that the variation of gene expression patterns is higher when there are more cells in the subgroup. The precision and recall rates of different methods are illustrated in Figures 2B,C. As DiffGE detects more differentially expressed genes, the recall rate is higher than those of the other three methods. When there are more cells in the subgroup, these methods detect more differential genes. Accordingly, the recall rates of these methods gradually increases. At the same time, DiffGE shows a higher precision. In detail, when the number of cells is >30% of the total number, the precision of DiffGE is higher than those of other methods. When the number of cells is 90% of the total set, these tested methods all have relatively high precision.

**Figure 3:**
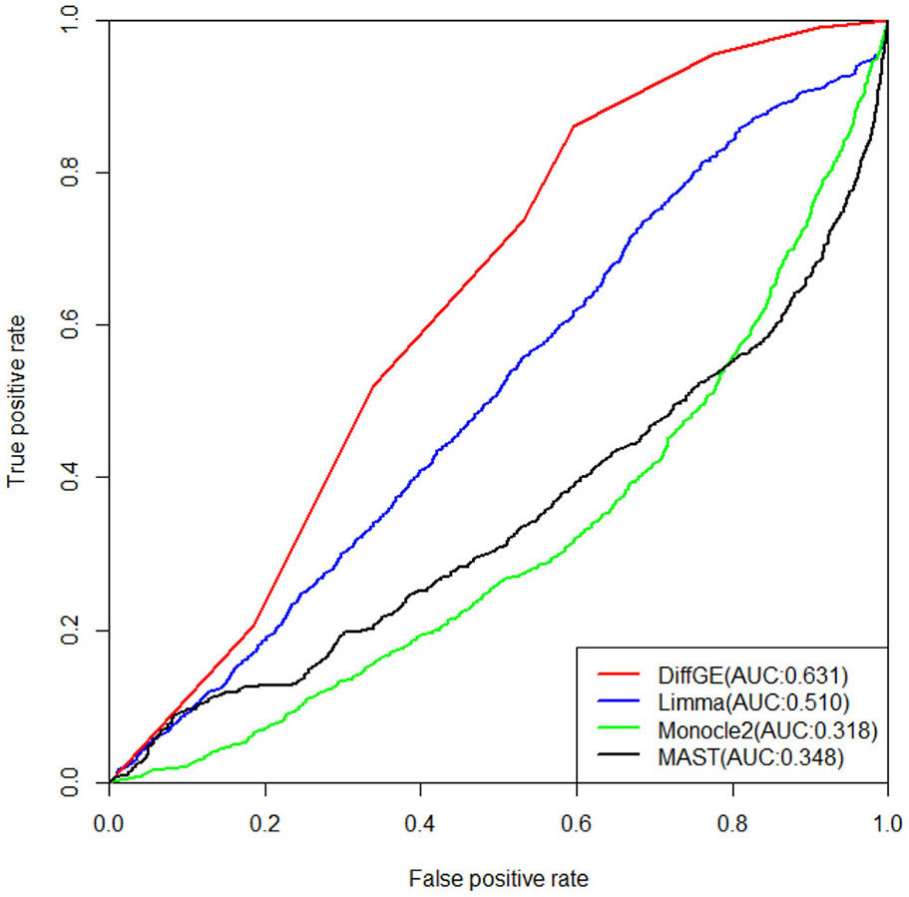
ROC curves of the three differential expression methods on the Islam dataset. Areas under the ROC curves (AUC) are shown in the parentheses.

Next, we validate the accuracy and robustness of DiffGE using the dataset from Islam et al. (2011). For validation of the detections, the results of Moliner et al. (2008) are used. The detections of different methods are evaluated by the areas under the receiver operating characteristic (ROC) curve (AUC). The ROC curves together with the corresponding AUC values of different methods are illustrated in Figure 3. For this dataset, we assess the detection based on the difference in expression of mouse ES and MEF cells. From the comparison result, we observe that our proposed DiffGE perform better than the other three methods. DiffGE produces a higher AUC value (0.631), followed by limma and MAST, while Monocle2 shows the overall lowest performance.

### 3.2. Identifying Highly Differential Gene Groups

To evaluate the performance of DiffGE, we apply it to the melanoma dataset. Based on the expression pattern, we first divide these genes into different gene groups. To identify those genes that are highly differentially expressed among different subtypes, we compute the network entropies of these gene groups among the six subtypes of melanoma. By ranking the gene groups with the calculated entropy, we focus on the top three gene groups, including 1,696 differential genes. For further analysis of the functional roles of the identified differential gene groups, we perform gene ontology (GO) enrichment analysis via DAVID bioinformatics resources (Huang et al., 2007), and summarize the involved key biological processes and pathways in Table 1. The significant enrichment lists are obtained with *p*-value < 0.001. Overall, for the gene groups with high network entropies, we observe that those genes exhibit enrichment for transcription regulation and cell development functions. For example, GO terms related to regulation, such as “Termination of RNA polymerase II transcription” and “Positive regulation of transcription” are enriched in the first and second differential gene groups; GO terms related to tumor, such as “Tumor necrosis factor-mediated signaling pathway” and “Apoptotic process” are enriched in these differential gene groups. The enrichment implys that the differential expression of cell-cycle and cell proliferation genes might be a key factor in tumor development. These results support previous findings that differential genes play critical roles in cell type specific regulation function.

**Table 1:** Functional enrichment of differential gene groups among different melanoma subtypes.

**Term type**	**Term name**	***P*-value**	**Term type**	**Term name**	***P*-value**
**GENE CLUSTER1**					
BP	mRNA splicing	2.83E-10	MF	Poly(A) RNA binding	1.60E-15
BP	T cell receptor signaling pathway	5.01E-07	CC	Nucleus	2.13E-07
BP	Cell-cell adhesion	1.15E-06	CC	Spliceosomal complex	2.80E-06
BP	Adaptive immune response	2.76E-06	MF	Protein N-terminus binding	9.87E-04
BP	Termination of RNA polymerase II transcription	5.37E-05	KEGG	Spliceosome	1.82E-05
BP	Apoptotic process	8.82E-04	KEGG	Viral carcinogenesis	4.31E-04
**GENE CLUSTER2**					
BP	Termination of RNA polymerase II transcription	1.26E-04	MF	Protein binding	3.74E-18
BP	Positive regulation of transcription	2.46E-04	MF	Poly(A) RNA binding	5.83E-12
BP	Tumor necrosis factor-mediated signaling pathway	8.93E-04	CC	Nucleoplasm	3.48E-15
BP	MRNA processing	4.95E-04	KEGG	Spliceosome	8.95E-10
**GENE CLUSTER3**					
BP	Oxidation-reduction process	4.13E-07	CC	Extracellular exosome	4.63E-13
BP	Negative regulation of apoptotic process	1.06E-04	MF	Protein binding	3.73E-09
BP	Translational initiation	4.27E-04	CC	Cell-cell adherens junction	1.35E-06
BP	Regulation of mRNA stability	6.09E-04	KEGG	Antigen processing and presentation	3.98E-04

**Figure 4:**
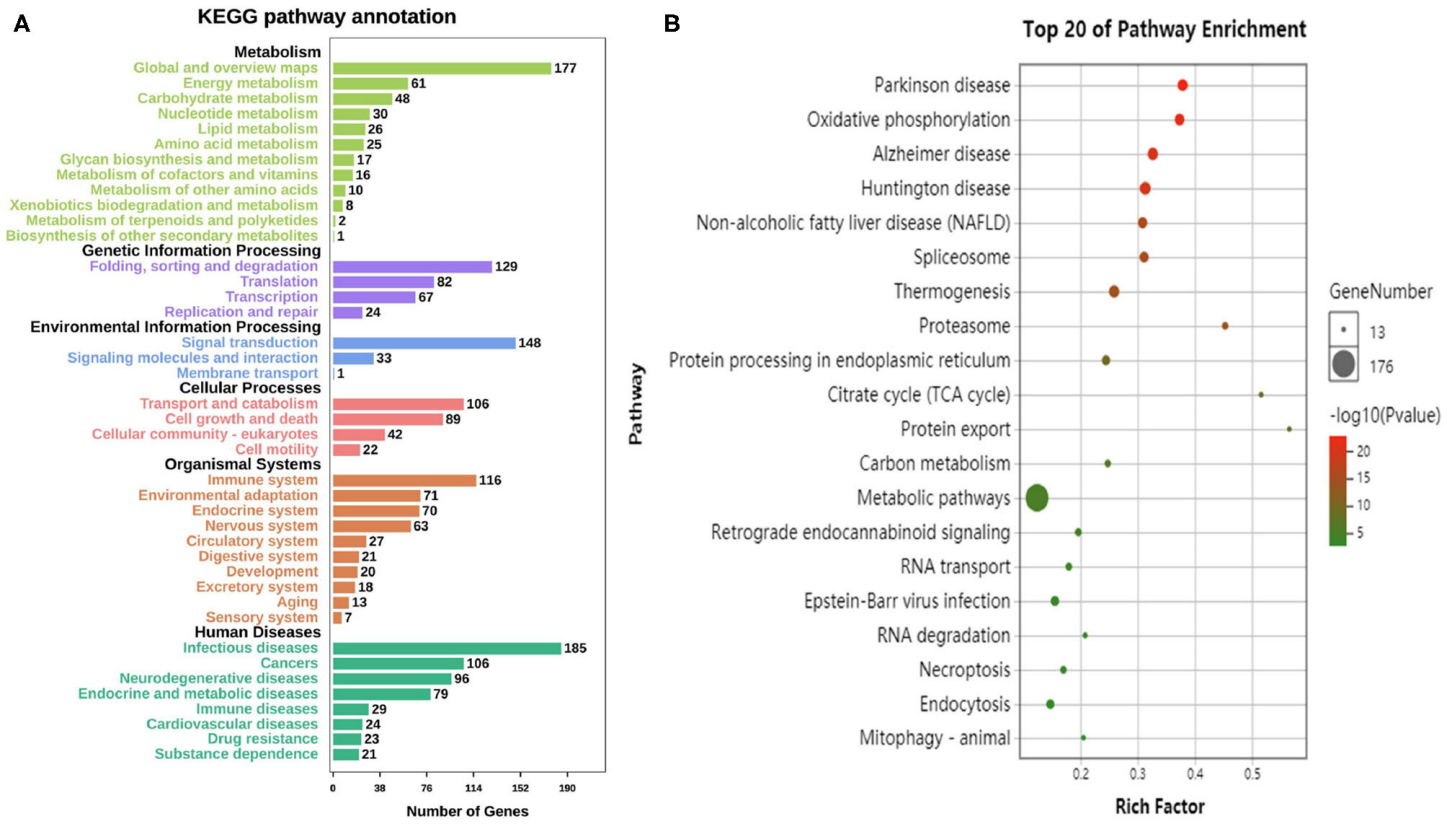
Enrichment analysis of differential genes. **(A)** The annotation results are classified according to the type of KEGG pathway. **(B)** The top 20 pathways of KEGG enrichments.

According to the KEGG database, the differential genes are enriched in 304 Pathways. The annotation results are classified according to the type of pathway (Figure 4A). The results show that the pathways of differential genes are closely related to metabolism, human disease, and organismal system. The KEGG pathway enriched bubble map can clearly show the significant enrichment pathway of differentially expressed genes. The abscissa indicates the enrichment factor. The larger the value, the more significant the differential genes are in the pathway. The size of the bubble indicates the number of genes. The larger the bubble, the more the number of genes are enriched in the pathway. The depth of the bubble indicates the level of significance, and the redder color indicates the higher the significance of enrichment to the pathway. The figure shows the top 20 pathways which the enrichment is most reliable (Figure 4B).

## 4. Discussion

Although the advent of single-cell RNA sequencing has provided new insights into cell dynamics, it has also brought new computational challenges. On one hand, the expression data obtained by scRNA-seq is relatively noisy, and then relevant models need to be considered. On the other hand, given a huge cell population, it is challenge to take on the task of unraveling cell heterogeneity. Analysis of differential gene expression is essential for almost all single-cell transcriptional studies. As previous studies show that genes in the same pathway tend to correlate in expression, the network structure of genes can boost differential expression analysis. In this study, we propose a new computational method, DiffGE, for identifying differential genes from scRNA-seq data. DiffGE extends the notion of local entropy to network entropy, which incorporates protein interactions to improve sensitivity and specificity of detecting differentially expressed genes from scRNA-seq data. It first groups genes, calculates the network entropy according to the PPI network of each gene group, and then detects highly differential genes groups by selecting gene clusters with higher network entropies. We evaluate the detection accuracy of the proposed method using three real scRNA-seq datasets and compare its performance with three widely-used differential expression analysis methods. The comparison results indicate that employing the gene interaction network can make a significant contribution to the accuracy and the proposed method has an powerful performance in detecting highly differential genes.

## Data Availability Statement

All datasets generated for this study are included in the article/supplementary material.

## Author Contributions

YG was responsible for the main idea, as well as the completion of the manuscript. SL have developed the algorithm and performed data analysis. GZ have coordinated the data pre-processing and supervised the effort. All authors have read and approved the final manuscript.

## Conflict of Interest

The authors declare that the research was conducted in the absence of any commercial or financial relationships that could be construed as a potential conflict of interest.
